# Autologous mesenchymal stem cell therapy for progressive supranuclear palsy: translation into a phase I controlled, randomized clinical study

**DOI:** 10.1186/1479-5876-12-14

**Published:** 2014-01-17

**Authors:** Rosaria Giordano, Margherita Canesi, Maurizio Isalberti, Ioannis Ugo Isaias, Tiziana Montemurro, Mariele Viganò, Elisa Montelatici, Valentina Boldrin, Riccardo Benti, Agostino Cortelezzi, Nicola Fracchiolla, Lorenza Lazzari, Gianni Pezzoli

**Affiliations:** 1 Cell Factory, Unit of Cell Therapy and Cryobiology, Fondazione IRCCS Ca’ Granda Ospedale Maggiore Policlinico, Milano, Italy; 2 Parkinson Center, Istituti Clinici di Perfezionamento, Milano, Italy; 3 Interventional Neuroradiology Unit, Fondazione IRCCS Ca’ Granda Ospedale Maggiore Policlinico, Milano, Italy; 4 Klinik und Poliklinik für Neurologie, Universitätsklinikum Würzburg, Würzburg, Germany; 5 Nuclear Medicine Unit, Fondazione IRCCS Ca’ Granda Ospedale Maggiore Policlinico, Milano, Italy; 6 Hematology and Transplantation Unit, Fondazione IRCCS Ca’ Granda Ospedale Maggiore Policlinico and University of Milan, Milan, Italy

**Keywords:** Progressive supranuclear palsy, Parkinson’s disease, Mesenchymal stem and stromal cells, Advanced therapy medicinal products, Cellular therapy

## Abstract

**Background:**

Progressive Supranuclear Palsy (PSP) is a sporadic and progressive neurodegenerative disease which belongs to the family of tauopathies and involves both cortical and subcortical structures. No effective therapy is to date available.

**Methods/design:**

Autologous bone marrow (BM) mesenchymal stem cells (MSC) from patients affected by different type of parkinsonisms have shown their ability to improve the dopaminergic function in preclinical and clinical models. It is also possible to isolate and expand MSC from the BM of PSP patients with the same proliferation rate and immuphenotypic profile as MSC from healthy donors. BM MSC can be efficiently delivered to the affected brain regions of PSP patients where they can exert their beneficial effects through different mechanisms including the secretion of neurotrophic factors.

Here we propose a randomized, placebo-controlled, double-blind phase I clinical trial in patients affected by PSP with MSC delivered via intra-arterial injection.

**Discussion:**

To our knowledge, this is the first clinical trial to be applied in a no-option parkinsonism that aims to test the safety and to exploit the properties of autologous mesenchymal stem cells in reducing disease progression. The study has been designed to test the safety of this “first-in-man” approach and to preliminarily explore its efficacy by excluding the placebo effect.

**Trial registration:**

NCT01824121

## Background

Progressive supranuclear palsy (PSP) is a rare form of parkinsonism with a prevalence of about 0.5 cases per 100,000 inhabitants and with an incidence of 5.3 new cases every 100,000 inhabitants [1,2]. Its etiology is unknown. From a pathological point of view, the disease consists in a neurodegenerative process that involves the basal ganglia, the brainstem, the prefrontal cortex and the cerebellum, with accumulation of a tau protein - hence the classification as tauopathy [3]. Onset typically occurs after 40 years of age. The symptoms include bradykinesia, proximal and axial rigidity and early postural instability. The key sign, which gives the disease its name, is the supranuclear paralysis of vertical gaze, followed by abnormalities of horizontal gaze. This sign usually appears three or four years after the onset of motor symptoms. The most disabling symptoms, especially in the early phases of the disease, are stiff, upright posture and abnormal gait with a very broad base associated with severe postural instability and frequent falls, especially backwards. The patient is confined to a wheelchair on average after 5 years of disease. Levodopa response is poor or absent [4-6]. Mean survival amounts to 7 years [7]. Early falls, speech and swallowing problems, diplopia and early insertion of a percutaneous gastrostomy are predictors of reduced survival [8].

The differential diagnosis between PSP and the other parkinsonisms is made according to clinical criteria [9]. Molecular biology studies have shown that the parkinsonisms share a common pathogenesis, namely the intraneuronal accumulation of misfolded proteins that cannot be removed normally. The misfolding is caused by structural abnormalities due to genetic mutation and/or exposure to environmental factors [10]. The syndromes have been classified according to the kind of misfolded proteins that accumulate: synucleinopathies, in which the main accumulated protein is alpha-synuclein, and tauopathies, in which it is protein tau [11]. Unfortunately the greater understanding of the parkinsonisms in terms of molecular biology has not resulted in the finding of a cure. At present all these movement disorders are incurable. However, symptomatic treatment is available. It consists mainly in dopaminergic treatment (levodopa and dopamine agonists), which controls symptoms for several years in Parkinson’s Disease (PD), whereas the response is generally poor and short-lived in the other syndromes [12]. Moreover, even in PD it is not effective on the most disabling symptoms, such as postural instability and freezing. Also surgical treatment is available. It consists in the implantation in strategic positions of electrodes for deep brain stimulation, which corrects electrical circuit imbalances occurring in circumscribed parts of the brain in PD patients [13,14]. However, this treatment is not suitable for the other syndromes, in which the neurodegenerative process is more extensive and general conditions deteriorate more rapidly.

### Summary of pre-clinical data to support the use of autologous MSC in PSP patients

Mesenchymal stem cells (MSC) are multipotent cells that can be isolated from many sources, including bone marrow (BM). Besides their in vitro and in vivo potential to transdifferentiate into several mesodermal lineages, their therapeutic relevance is mostly due to their immunosuppressive and anti-inflammatory properties [15]. Other paracrine actions of MSC have been claimed to act in several animal models of diseases and also in preliminary clinical trials [16-18]. Indeed, the real MSC trans-differentiation capacity seems to have limited clinical relevance, with the exception of bone differentiation that is currently exploited in different orthopaedic trials [19]. The main expected pharmacological effect of BM MSC on dopaminergic neurons that support their use in PSP is the potential to regulate cell differentiation and function by reducing oxidative stress and apoptosis. Regarding this potential mechanism of action, there are several experimental evidences of the neurotrophic effect of MSC that result in the reduction of tissue damage and neuronal loss. The brain derived neurotrophic factor (BDNF) and the glial derived neurotrophic factor (GDNF) have been recently identified as the putative mediators of this effect. BDNF is a protein belonging to the neurotrophine family that promotes neuronal growth, differentiation and survival in different areas of central and peripheral nervous system. The molecular pathway that mediates the BDNF effects on cell survival is well known and documented [20]. Briefly, the homodimeric BDNF induces the dimerization of the thyrosin-kinase receptor B, the binding of ATP to the intracellular ATP-binding loop, and in turn the stimulation of the kinase activity. The autophosphorylation of the tyrosine triplet in the kinase domain is a prerequisite for further phosphorylation steps (via Src homology 2/collagen-related protein and PIK3and MAPK pathways) that finally mediate the effects of the neurotrophin on neuronal survival, differentiation, and gene expression as well as acute effects on synaptic transmission [21].

Also the molecular basis of GDNF mechanism of action is well know and consists essentially in its interaction with the GDNF-family receptor-alpha (GFRa1), on the activation of the Ret tyrosine-kynase [22] and on the PLCγ, PI3/Akt and MEK-ERK1/2 pathway [23]. Nevertheless, it is also known that the GDNF- GFRa1 complex can work independently from Ret activation by activating the Src family and consequently the c-AMP-response element binding protein (CREB) [24,25]. All these mechanisms have a central role in regulating the survival of dopaminergic neurons [26]. The precise balancing of these signals results in the definition of neuronal cell fate in response to different noxa.

**Figure 1:**
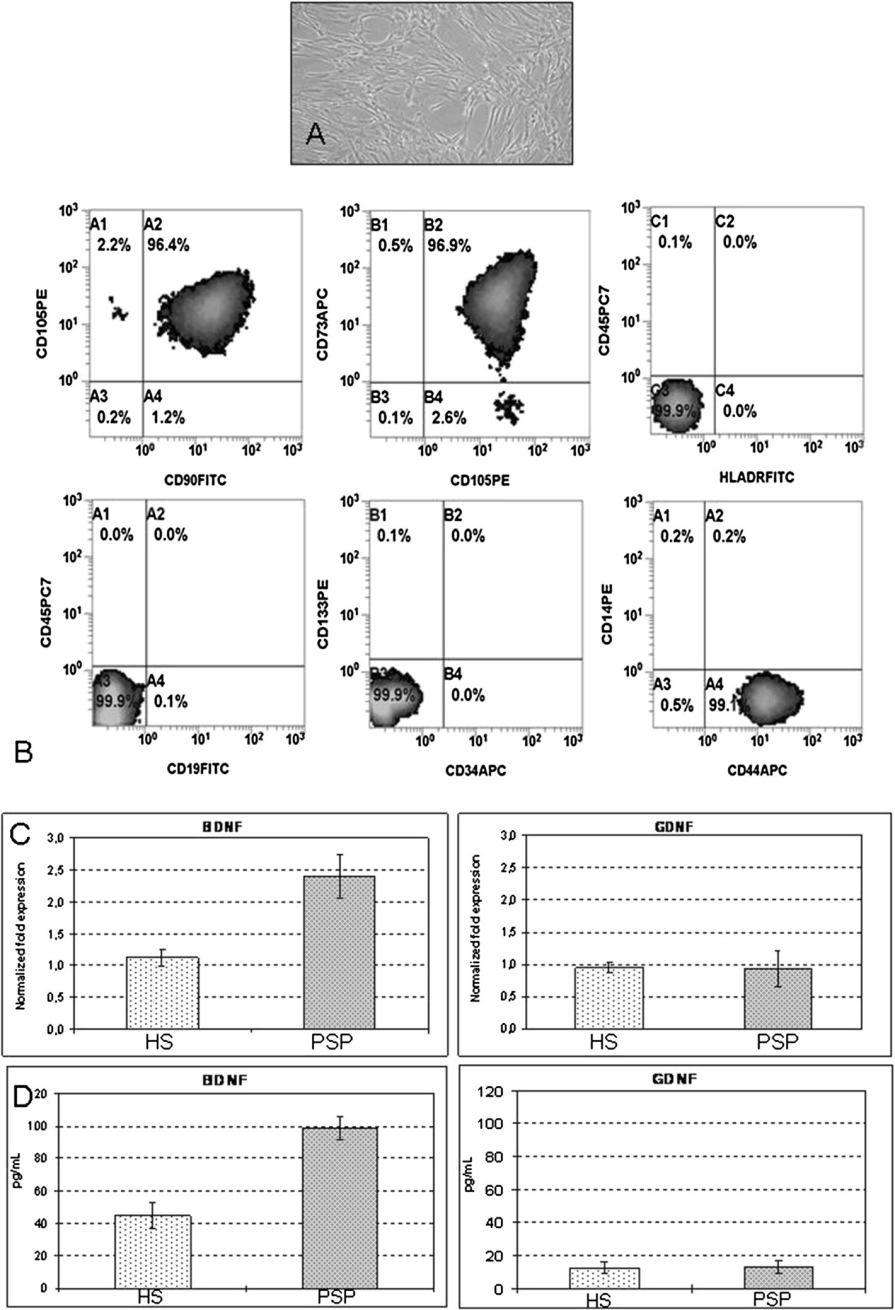
**Pre-clinical data.** Bone marrow mesenchymal stem cells from patients affected by progressive supranuclear palsy have the typical spindle-shaped morphology **(A)**, are positive for mesenchymal-specific antigens to an extended flow-cytometric analysis **(B)** and express BDNF and GDNF as those from healthy subjects, as demonstrated by real time PCR **(C)** and ELISA **(D)**. BM MSC: bone marrow mesenchymal stem cells; PSP: Progressive Supranuclear Palsy; HS: healthy subjects. The results of flow cytometric analysis, Elisa and PCR are expressed as mean (±SD).

In order to establish the role of the hypothesized mechanism of action, the ability to synthesize and secrete BDNF and GDNF by MSC from PSP patients and healthy donors has been evaluated, by real–time PCR and by ELISA. The results are shown in Figure 1 and they demonstrate that MSC from PSP are able to produce as main neurotrophine as MSC from healthy donors. For that reason, BDNF and GDNF secretion from MSC will be measured during the clinical protocol as potency assay. The use of cells instead of the simple administration of synthetic proteins has several advantages. First of all endogenously produced neurotrophines are in principle fully bio-available since MSC could be able to vehicle directly the neurotrophines to the damaged tissue. In this regard, it has been already demonstrated that MSC are able to overcome the brain–blood barrier [27,28]. MSC may also act by releasing extracellular vesicles, including exosomes and microvescicles, which transport lipids and different mRNA functional transcripts, microRNA, long non-coding RNA and occasionally genomic DNA and therefore they could be able to transfer genetic information that may induce transient or persistent changes in the recipient cells [29].

## Methods/design

The current trial is a prospective, randomized, sham-controlled, phase I clinical study to evaluate the safety and efficacy of autologous mesenchymal stem cell intra-arterial infusion in patients with PSP. The protocol has been authorized by the local Ethics Committee of Fondazione IRCCS Ca’ Granda Ospedale Maggiore Policlinico of Milano, Italy, and by the National Competent Authority for phase I cell therapy protocol at Istituto Superiore di Sanità.

### Primary and secondary objectives

The primary objective is to assess the safety of autologous MSC therapy in patients with PSP in a “first-in-man” context.

The secondary objective is to assess the efficacy of autologous MSC therapy in patients with PSP in terms of stabilization or improvements in motor function, neuropsychological parameters and neuroimaging findings.

### Exploratory objectives

Exploratory objectives will help to identify the mechanisms underlying the effect of MSC on neurodegeneration. Whit this aim, the ability of MSC to *in vitro* rescue 6-OHDA damaged neural cell lines and to synthesize and secrete neurotrophines will be measured to determine if these factors are related to the clinical response.

### Study design

All patients over the age of 40 years with diagnosis of “probable progressive supranuclear palsy - Richardson’s disease subtype” according to current diagnostic criteria [4; 9] are eligible (Table 1).

**Table 1:** Inclusion and exclusion criteria

***Inclusion criteria***	-Diagnosis of ’probable Progressive Supranuclear Palsy - Richardson’s disease subtype’ according to current diagnostic criteria [4; 9], including akinetic-rigid syndrome;
	-Age at onset ≥ 40 years;
	-Disease duration 12 months to 8 years;
	-Supranuclear ophthalmoplegia;
	-Postural instability or falls within 3 years from disease onset;
	-Positive MRI for PSP criteria (Quattrone et al., [30]);
	-Stable pharmacological treatment for at least 90 days;
	-Lack of response to chronic levodopa (at least 12-month treatment);
	-Able to stand in upright posture without assistance for at least 30 seconds;
	-Written informed consent (including video taping).
***Exclusion criteria***	-Idiopathic Parkinson’s disease;
	-Cerebellar ataxia;
	-Symptomatic autonomic dysfunction;
	-Evidence of any other neurological disease that could explain signs;
	-History of repeated strokes with stepwise progression of parkinsonian features;
	-History of major stroke;
	-Any history of severe or repeated head injur;
	-A history of encephalitis;
	-A history of neuroleptic use for a prolonged period of time or within the past 6 months;
	-Street-drug related parkinsonism;
	-Significant other neurological disease on CT-scan/MRI;
	-Oculogyric crises; -major signs of corticobasal degeneration;
	-Signs of Lewy body disease;
	-Other life-threatening disease likely to interfere with the main outcome measure;
	-Any clinically significant laboratory abnormality, with the exception of cholesterol, triglycerides and glucose;
	-Renal failure (serum creatinine >300 mM/L);
	-Transaminase elevation > twice upper limit of normal;
	-Any concomitant disorder associated with bone marrow function impairment;
	-Any concomitant disorder that requires chronic treatment with immunosuppressors, anti-inflammatory agents, and/or growth factors;
	-Dementia (MMSE < 24 according to Folstein 1975 or defined according to DSM-IV TR criteria);
	-Any other disorder that could interfere with the evaluation of treatment or that could make intra-arterial infusion inadvisable;
	-Any other features that, according to the investigator, could reduce adherence to protocol procedures or prevent rapid access in case of emergency;
	-Women of child-bearing age;
	-Participation in another clinical trial with experimental treatment in the last 30 days;
	-Brain MRI evidence of severe vascular abnormalities, space-occupying lesions or normal pressure hydrocephalus.

The first 5 patients are treated in an open phase with autologous MSC therapy with the same procedures as for the randomized phase.

After these first 5 patients have been followed-up for a minimum of 2 months from the cellular infusion, the ISS Data Safety Monitoring Board will review the safety data prior to open the accrual of the subsequent randomized controlled phase.

In the randomized phase the patients undergo to:

–immediate autologous MSC therapy followed by delayed sham or

–immediate sham followed by delayed autologous MSC therapy.

**Figure 2:**
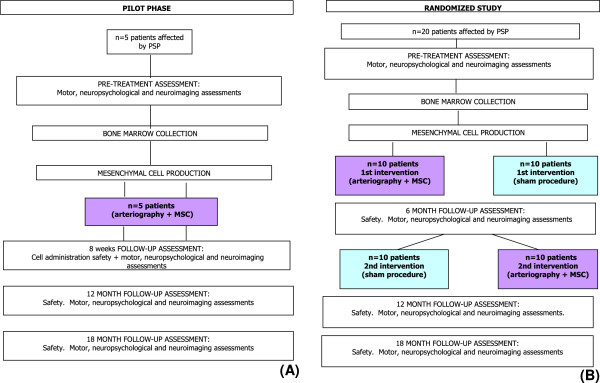
**Study design. A)** Pilot phase; **B)** Randomized study.

The delay amounts to 6 months and all patients will be followed-up for at least 12 months after MSC therapy, so the total duration of the study is 18 months. The study design is shown in Figure 2.

### Bone marrow collection and MSC isolation

Bone marrow is aseptically drawn by qualified medical staff at the Bone Marrow Transplantation Centre - Fondazione IRCCS Ca’ Granda Ospedale Maggiore Policlinico Milano according to standard procedures. The maximum quantity of bone marrow to be collected is 30 ml. The isolation of BM MSC is performed under Good Manufacturig Practices (GMP) conditions as requested by European Regulations for cell-based advanced therapy medicinal product (ATMPs) in the “Cell Factory” Laboratory of Fondazione IRCCS Ca’ Granda Ospedale Maggiore Policlinico Milano. The “Cell Factory” was the first public Italian hospital-based facility to receive authorization for the production of ATMPs (Agenzia Italiana del Farmaco – AIFA – authorization n°120/2007 and subsequent confirmations, the last in 2013). The procedures for BM MSC aseptic production and quality control have been developed by the authors. Briefly, unprocessed BM is directly seeded in alpha Modified Eagle Medium supplemented with 10% FBS at the concentration of 50,000 total nucleated cells (TNC)/cm^2^ in Cell Stack Chamber system (Corning, Lowel, MA). After 72 hours, non-adherent cells are removed by washing with PBS (Macopharma, Mouvaux, France) with complete medium change. Medium changes are also performed twice a week. On day 14 (±3) MSC at P0 are detached using 25 mL/layer of TrypLE- Select (Gibco-Life Technologies, Carlsbad, CA, USA) and re-seeded in the same culture conditions at the concentration of 4000 MSCs/cm2. The culture is stopped at 28 days (±3) of culture (passage 2) and the cells are re-suspended in a solution containing normal saline solution with human serum albumin (Kedrion, Castelvecchio Pascoli, Lucca, Italy) 10% (vol:vol) and DMSO (Bioniche Lifesciences, Inc., Belleville, ON, Canada) 10% (vol:vol). The cell product is cryopreserved using a controlled-rate freezer (Nicool Plus, Air Liquide) programmed to freeze at −1 C/ min and is stored in the vapor phase of liquid nitrogen in bags (CryoMACS Miltenyi, Teterow, Germany). The day of the infusion, the cells are thawed at 37°C and resuspended (1:2) in normal saline solution with human serum albumin (Kedrion) 10% (vol:vol) and ACD-A 12% (Fresenius Kabi, Bad Homburg, Germany). Finally, volume is adjusted to 200 mL with normal saline solution alone.

### Administration of MSCs

Patients undergo neuroleptoanalgesia and are monitored by an anesthesiologist. MSCs are administered by intra-arterial route, as already described [28], with modifications according to local equipment and local standards: with Seldinger technique, catheterization is carried out via the right common femoral artery (or the left one in the event of difficulty in achieving arterial access) using a 6 F Ultimum EV (St Jude Medical, Minnetonka, MN, USA) introducer and a 5 F Hinck or Simmons (Terumo Europe NV, Leuven, Belgium) diagnostic catheter. An angiographic study of the cervical and intracranial arteries is performed, with the support of a 0.035 inch, 150 cm long hydrophil guide (Terumo Europe NV, leuven, Belgium). Subsequently, with or without an exchange maneuver, using a 260 cm Starter exchange (Boston Scientific, Natick, MA, USA), a Mach 190 cm catheter guide (Boston Scientific, Natick, MA, USA) is used, after intravenous administration of a bolus of heparin sodium (3,000 to 5,000 IU according to body mass) to reduce the risk of thromboembolism. The catheter guide will be positioned in the widest vertebral artery. Subsequently, the main catheter is placed in both internal carotid arteries and a microcatheter is moved forward up to the widest vertebral artery.

The MSC are then injected into the various districts with an automated peristaltic pump, via the microcatheter. Should any ulcerated atherosclerotic plaques be found at carotid bifurcations, therapy is injected proximally to the lesion. Once therapy administration has been completed, MRI of the brain is performed with two sequences, FLAIR and DWI. Patients are closely monitored for 3 hours after the procedure and then moved to the Neurosurgery Unit, where they spend the next 24 hours. Provided that the procedure was uneventful, they are discharged the day after the cell injection. Patients undergoing the sham procedure are monitored in the same way.

### Motor status assessment

The patients undergo neurological examinations designed to assess motor function using the following scales:

Unified Parkinson’s Disease Rating Scale (MDS-UPDRS) total and motor score [31];

Hoehn and Yahr staging [32];

PSP rating scale [33] to specifically rate PSP severity;

SEADL (Schwab England Activities of Daily Living);

CGI (Clinical Global Impression for disease severity) [34];

If the patient is taking dopaminergic therapy, the neurological assessment is performed twice: in OFF upon wakening and in ‘ON’ after taking therapy in the morning by the same neurologist.

After patient’s consent, the entire neurological examination is video-recorded so that the evaluation can be performed again by assessors blind to allocation.

### Multifactorial movement analysis

Patients perform four tasks:

(1.) upright standing;

(2.) starting to walk;

(3.) linear free gait (with and without a cognitive task);

(4.) gait along a curvilinear trajectory.

Tests are carried out in the order listed above as already described with minor modifications [35], if possible given the clinical status of the patient, before and after MSC therapy. Each test requires 4–8 walks, at intervals of about 5–10 minutes, on a platform about 10 meters long at a comfortable speed. The cognitive task consists in a calculation (counting backwards, starting from 100 and subtracting 7 at a time). The patients are not corrected or stopped if they make mistakes in calculating. The curvilinear test consists in straight walking for 2 meters towards the center of the platform and then a curvilinear trajectory with an angle of 90° for another 2 meters. Data are collected with a SMART optoelectronic movement analysis system (BTS SpA, Italy) equipped with: (i) optoelectronic system (six cameras with 60 Hz acquisition frequency) to establish the position of 29 reflecting markers applied onto the skin of the patients at anatomical landmarks (ii) three dynamometric platforms (KISLER, GmbH, Winthertur, Switzerland) with 960 Hz acquisition frequency to record ground reaction forces; (iii) TELEMG telemetric electromyograph (BTS SpA) and a FreeEMG wireless electromyography to measure muscular activity of tibialis anterior and soleus bilaterally.

### Neuropsychological assessment

The following tests are performed:

–Mini Mental State Examination (MMSE) [36]

–Neuropsychological measures: verbal comprehension; perceptual organization, immediate memory (story, design, word list learning), delayed memory (story, design, word list recall), word list recognition, language (confrontation naming, category fluency, letter fluency, auditory comprehension), attention/concentration, visuospatial ability (block design), processing speed, executive functioning (cognitive flexibility).

### Quality of life

Quality of life is assessed by asking the patients to fill-in the PDQ-8 questionnaire [37] at baseline, and 6 and 12 months after MSC therapy.

### Neuroimaging

All patients perform a longitudinal neuroimaging assessment at baseline and 6, 12 and 18 months after MSC therapy, using striatal dopamine transporter Single Photon Emission Computed Tomography (SPECT) and Positron Emission Tomography (PET) using a tropanic tracer labeled with Iodine-123 (FP-CIT) for SPECT imaging and Iodine-124 (Beta-CIT) for PET/TC imaging. SPECT studies are carried after intravenous administration of 110-140 MBq of ^123^I-FP-CIT (Datscan®, GE-Health, Amersham, UK) performed 30–40 minutes after thyroid blockade (10–15 mg of Lugol solution per os) in all subjects. ^18^ F-fluorodeoxyglucose positron emission tomography (FDG-PET/TC) is performed 4–6 hours and 20–24 hours after intravenous injection of 18–30 MBq of ^124^I- Beta-CIT. All patients undergo ^18^ F-fluoro-2-deoxy-D-glucose positron emission tomography scanning (FDG-PET) at rest, after intravenous injection of 285 to 296 MBq.

### Safety

Toxicity is evaluated following the National Cancer Institute Common Toxicity Criteria Manual (CTCAE v 4.0) [38]. At screening demographic information, medical history and specific history of PSP (age at onset, signs and symptoms, previous investigations, with particular reference to neuroimages, treatment) are collected. Patients are monitored closely after the MSC/sham procedure for 3 hours at the day-hospital of the Diagnostic and Interventional Neuroradiology Unit and subsequently at the ward of the Neurosurgery Unit until discharge the next day. A general medical examination and routine laboratory tests are performed at every visit, plus any further investigations that are deemed necessary according to the findings of the general medical examination.

### Statistics

#### General considerations

As noted above, the safety of cellular therapy administration in the first five patients will be reviewed by the Data Safety Monitoring Board. These reviews will be shared with our institutional IRB. If the treatment appears safe, as determined by the Data Safety Monitoring Board with institutional IRB agreement, the protocol may be reopened to treat a total of 20 patients.

For statistical analysis of efficacy endpoints, descriptive statistics are computed for variables of interest (mean and SD for continuous variables and frequencies for categorical variables). Test results are summarized over time using appropriate graphical or tabular formats. The non parametric pair wise Wilcoxon rank sum test will be used to investigate each variable within subject and between the same subject at different follow-up (e.g. pre- vs. post-MSC, pre- vs. post-sham, etc.). The use of a non-parametric test has been considered the most appropriate since the distribution of the variable is not known and the number of observations is relatively small. For each time point, the test will be performed with 20 paired values (the values before and after MSC therapy in the 10 subjects allocated to immediate treatment plus the 10 subjects allocated to the delayed treatment).

P-values < 0,05 will be considered statistically significant. Additional summary statistics are also considered as appropriate to the data.

#### Safety data

A descriptive analysis of all AEs and ADRs will be provided.

The tabulations of laboratory data will be provided together with the normal ranges of the laboratory, highlighting any values that are out of range.

#### Motor function

A descriptive analysis of the course of the main endpoints will be provided:

–change in total, ADL, motor and part IV UPDRS score;

–change in Hoehn & Yahr stage;

–change in PSP rating scale (cognitive disturbances, bulbar functions, limb function, ocular motor function);

–change in SEADL;

–CGI score.

#### Multifactorial movement analysis

The data related to each of the four tests will be normalized and the mean of the values related to each variable recorded during the 8 walks will be calculated. The data before and after MSC therapy will be compared using Wilcoxon’s signed rank test for matched pairs, considering the patients before and after treatment as a pair (the results at each follow-up visit will be assessed separately). Moreover, the results obtained in PSP patients will be compared with those obtained in the group of age-matched healthy volunteers using the non-parametric Mann–Whitney U Test.

#### Neuropsychological assessments

A descriptive analysis of the course of the main endpoints (change in each of the test scores vs baseline will be provided).

#### Neuroimaging assessment

The main neuroimaging parameter will be:

–specific striatal uptake of the labeled ligand for SPECT analysis; this parameter measures the striatal density of dopamine transporters

–normalized labeled ligand subcortical and cortical uptake for PET analysis; this parameter measures normalized regional cerebral flow/glucose metabolism in the gray matter of the brain.

The analysis will be performed using ANOVA to assess changes in cerebral volume by SPM software. Significance will be set at p<0.01.

### Translational endopoints

#### In vitro model of tissue damage and repair

In our laboratory we set up an in vitro assay to assess the effect of MSC on neural cell damage and repair. Human neuroblastoma cell line SK-N-BE, cultured in appropriate medium, is damaged using 6-OHDA. After 24 hours, the cells are washed and MSC from PSP patients and, in parallel, from healthy subjects are added in no-contact co-culture using Transwell® Permeable supports. At different time points (0, 48, 72 and 144 hours) the viability of SK-N-BE cells is checked by quantification using 3-(4,5-dimethylthiazole-2yI)-2,5-diphenyltetrazolium bromide (MTT assay). In addition, at all the above defined time points, the supernatant are collected and stored at −80 C for the ELISA quantification.

#### Neurotrophine produced by MSC

In order to investigate the role of neurotrophines secreted by autologous MSC from PSP patients as mediators of their neuro-rescue potential, the production of multiple neurotrophic factors will be evaluated throw a multiplex sandwich ELISA (matrix metalloproteinase 2/3/9/13 - MMP 2/3/9/13; BDNF; GDNF; neurotrophin 3 - NT-3; basic nerve growth factor - BNGF, ciliary neuronotrophic factor - CNTF) on MSC supernatants and a quantitative RT-PCR analysis (for BDNF and GDNF) on MSC RNA extract.

### Innovation

The proposed first-in-man study has several innovative characteristics. First, we demonstrated that it is possible to produce an advanced therapy medicinal product, manufactured in compliance to GMP rules from the bone marrow of patients affected by PSP and that this product has the same safety profile as that produced from healthy donors. Second, we translated the concept of using MSC as a cell-based delivery system not only for neurotrophic factors, but also for different types of nucleic-acid-containing vesicles, able to transiently or permanently transfer genetic information to the surrounding cells. Moreover, the presented study aims to document the putative effect of cell therapy by combining clinical evaluation and multifactorial movement analysis. We have therefore established a multidisciplinary platform to study the effect of cell-based advanced therapy that can be applied not only to PSP patients, but also to other neurodegenerative disorders (e.g. MSA, PD, etc.).

## Discussion

Cell-based advanced therapy is a novel and extremely promising option to cure otherwise untreatable neurodegenerative diseases but there are still several bottle necks that slow the progression and the widening of clinical trials in this context. Mainly, despite the overall positive results reported in several small studies in PD and MSA, there is a lot of uncertainness regarding the real capacity of MSC to reach the brain, the selectivity of their targets and their real mechanism of action. Moreover, in PD as well as in atypical parkinsonisms no validated biomarker is available to follow-up the neuroprotective effect of putative disease-modifyng treatments. So, why are we performing this trial? First: there are several positive results in other no-option parkinsonisms as well as in PD that deserve to be confirmed also in other pathologic context like PSP. Therefore, even thought the primary objective of this protocol is to demonstrate the safety of autologous MSC intra-arterial administration in subjects affected by PSP, we have designed a sham-controlled study, with the aim to document efficacy as well. Second, we would like to indirectly get information on the putative neuroprotective mechanism of action of MSC in PSP by comparing the in vitro results of the proposed potency assay and the clinical outcome. The biological hypothesis underlying the rationale of the proposed protocol is that MSC can reduce the neural cell loss in PSP by reducing cell apoptosis and the detrimental consequence of oxidative stress on neural cell homeostasis. Whit this premises, it should be clear to the reader that, the objective of making neurons from stem cells is out of the scope of our approach. Nevertheless, the story of replacing lost neurons in parkinsonisms by transplantation has been pursued for years. At first tissue was transplanted. In the 1980s autologous adrenal gland medullary tissue was transplanted first in animals and then in humans. Significant clinical improvements were achieved, but common and even serious postoperative complications induced clinicians to abandon this option [39-44]. In the 1990s non-autologous fetal neuron transplantation was attempted. Clinical benefits were modest and confined to younger patients (<60 years), and complications occurred i.e. the appearance of postoperative dyskinesias that in a few cases were severe and refractory to treatment. PET investigations suggested that these cases were due to an imbalance in the dopaminergic circuits induced by the transplanted cells [45,46]. Recent in vivo brain imaging findings in two patients, who had exhibited major motor recovery after transplantation, suggested that the cause of the dyskinesias was a serotonergic hyperinnervation [47]. An alternative explanation is that an immune response was responsible for both the appearance of dyskinesias and the modest extent of the therapeutic benefits [48]. Furthermore, the autopsies of subjects who had received fetal mesencephalic dopaminergic neurons disclosed the presence of Lewy bodies, raising the possibility of host-to-graft disease propagation. Thus, the prospects for dopaminergic fetal neuron transplantation in PD do not appear to be promising [49]. Autologous stem cell transplantation has been proposed for the most common form of parkinsonism, PD [50]. This proposal overcomes the ethical reservations related to the use of fetal cells or embryonic stem cells, and removes the risk of transplant rejection. Even thought the ability of different bone-marrow derived stem cells to migrate into the brain and to differentiate into cells bearing neural markers have been postulated even in vivo [51-53], the proven evidence of the so-called neural plasticity have never been demonstrated. Indeed, in the last years the attention of most investigators has been addressed to study the potential of BM mesenchymal stem cells to positively influence neural cell survival and to reduce cell apoptosis [54-56]. MSC have proved to be able to produce different kinds of growth factors that increase neuronal survival, to possess immunoregulatory properties able to influence inflammatory conditions and to migrate to damaged tissue areas where they might contribute to counteract neurodegeneration. What is more, they can be easily harvested from the bone marrow of the patients, easily expanded on a large scale for autotransplantation, and administered to patients via various routes [57]. MSCs have been isolated from PD patients and they do not differ from those of healthy subjects in terms of phenotype, morphology and ability to differentiate [58]. Furthermore, it has been demonstrated that MSCs exert neuroprotective effects on dopaminergic neurons both in vitro and in animal models of PD (rats given the toxin MG-132 or 6-OHDA), mediated by anti-inflammatory activity and the secretion of growth factors [59-62]. Of particular interest is the fact that in the rats given MG-132, a proteasome inhibitor, MSCs also reduced the accumulation of polyubiquinated proteins, a finding that suggests that they also contribute to correct proteasome dysfunction, which is believed to play an important role in the pathogenesis of PD [60].

Autologous MSC therapy would therefore appear to be an important candidate for the development of a parkinsonism-modifying therapeutic strategy.

To our knowledge, four pilot clinical trials have been published on the use of autologous stem cell transplantation in patients with primary parkinsonism. Two studies [63,64] have been conducted in patients with multiple system atrophy (MSA). The first one was an open-label, controlled trial designed to assess the feasibility and safety of MSC therapy by intra-arterial and intravenous route in which eleven MSA patients were infused MSC therapy through the internal carotid artery and the proximal portion of the vertebral artery once and by intravenous route thereafter once a month for three months. Treated patients were compared to 18 untreated control MSA patients. Follow-up was continued up to one year after the beginning of treatment and consisted in neurological examinations using the unified MSA rating scale scores (UMSARS). In addition, 5 treated patients and 10 untreated patients underwent brain metabolism imaging using PET and ^18^ F-fluoro-deoxyglucose (FDG). After 12 months mean total UMSARS score was similar to the score at baseline in the treated group (functional stabilization), whereas it had worsened in the control group (p = 0.002). Moreover, treated patients showed increased brain metabolism by means of FDG-PET, while the untreated group had decreased uptake. The most significant adverse events reported was the occurrence in 7 patients of small spotty lesions on MR images that were considered to be asymptomatic microemboli, a frequent complication of catheterization techniques. The same group recently published the results of a second protocol, in which thirty-three patients with probable MSA-C were randomly assigned to receive MSC via intra-arterial and intravenous routes or placebo. The primary outcome was change in the total UMSARS scores from baseline throughout a one-year follow-up period between groups. In this study, the MSC group had a smaller increase in total and part II UMSARS scores compared with the placebo group. Cerebral glucose metabolism and gray matter density were more extensively decreased in the cerebellum and the cerebral cortical areas, along with greater deterioration of frontal cognition in the placebo group compared with the MSC group. No serious adverse events directly related to MSC treatment were recorded. However, intra-arterial infusion resulted again in small ischemic lesions on MR. In another open-label clinical trial [65], autologous BM MSCs were transplanted into a sublateral ventricular zone of the brain by stereotaxic surgery in 7 male patients aged 22 to 62 years with advanced PD. The diagnosis was based on the presence of the classical symptoms and a good response to levodopa. The patients were followed up for 10 to 36 months. Three of the patients experienced motor improvement compared to baseline; the mean extent of improvement in UPDRS score was 22.9% in OFF and 38% in ON. Two of the patients were able to reduce the dosage of their antiparkinson medications. No serious adverse events occurred. MRI imaging did not disclose any significant changes. The last study [28] was an open-label clinical trial conducted by interventional radiologists, who subjected 53 patients with a diagnosis of PD made according to UK Brain Bank criteria to intraarterial autologous implantation of mononuclear cells from bone marrow. The cells were introduced by intraarterial catheterization, infusing the cells into the posterior part of the circle of Willis, from which the perforating arteries that irrigate the basal nucleus and the substantia nigra originate. Four patients received a second implant. None of the patients had major complications. They experienced major significant changes in median disease severity scores: UPDRS, Hoehn & Yahr, Schwab & England and Northwestern University Disability Scale (NUDS). In eight patients follow-up MR spectroscopy revealed mean improvements in n-acetylaspartate/creatine ratio. These studies demonstrated the feasibility of autologous cell transplantation in patients with parkinsonism, but they did not demonstrate efficacy, because their design did not ensure objective measurements. Also, clinical improvement could have been influenced by the beliefs of patients and investigators alike as they were open-label studies. Neuroimaging changes could not be easily dismissed, but they were available only in a few patients and did not focus on the kind of damage that is seen in parkinsonism. A search in the WHO worldwide clinical trial database on August 1, 2013 disclosed 390 studies assessing MSC therapy. Out of these only other three studies are ongoing in patients with Parkinson. To our knowledge this is the first time that autologous MSC therapy is given to PSP patients.

In this kind of cell therapy protocols, a crucial question is how to fix the optimal cell dose to be administered to the patients and which is the best administration route, considering together the safety aspects and the efficacy objectives. When the protocol was conceived, the dose to be given was established based on several considerations. In particular, although there were data showing that MSCs in PD patients do not differ from those of healthy subjects in terms of phenotype, morphology and ability to differentiate into other cells [58], a preliminary study was conducted in PSP patients, to check the bone marrow function and MSC yield and to establish how many cells could be realistically produced for administration. The elements that have been used for the definition of the cell dose were the quaantity of MSCs that was obtained from maximum 30 mL of bone marrow and the cell dose that was given in the three protocols that were previously performed with MSC in parkinsonisms (1-2×10^6^/kg). Thus, the cell dose was fixed at 1.5 ± 0.5 ×10^6^/kg. Regarding the route of administration, several possibilities were considered. Systemically injected MSC undergo intra-pulmonary cell trapping [66] and therefore only a limited amount of cells might home to the brain. Stereotaxic-guided intra-striatal implantation was excluded in consideration of the pathologic characteristics of PSP that has a much wider distribution compared to classic Parkinson disease and for safety concerns, since there are several evidences that intra-striatal cell administration may cause harm and reduce efficacy by evoking a local cellular immune response [67]. Therefore, we decided to use superselective arterial catheterization to implant stem cells throw the arteries that feed the brain regions affected by PSP to release in situ the highest concentration of MSC. This technique has been used in the three out of four previous clinical trial in parkinsonisms, with no major adverse events out of the report of asymptomatic microembolism. To minimize this risk, several precautions have been taken in our protocol during the preparation of the cell product such as dilution of the cells to less than 1 × 10E6 cells/mL and the addition of an anticoagulant (ACD-A) to the solution in which the cells are re-suspended before infusion.

In this study a “pure” control group treated with placebo solution administered by the same route in a double-blinded manner is missing since this option was considered not acceptable by the ethical point of view by the investigators, given the potential harm of a cerebral artery catheterization procedure that is not justified by a potential benefit. Nevertheless, the patients enrolled in the control harm receive a simulated arterial catheterization and administration procedure and the neurologist and the neuro-radiologist who perform the follow-up evaluation are blinded. In this way, we exclude the placebo effect with the best precision.

In conclusions, the presented protocol is the first attempt to understand if MSC can be safely administered and can exert a beneficial effect in PSP patients. We believe that the results of this trial will help to improve the knowledge around the neuroprotective properties of MSC that might be exploited in other several neurodegenerative disorders.

## Abbreviations

BDNF: Brain-derived neurotrophic factor; BNGF: Basic nerve growth factor; CNTF: Ciliary neuronotrophic factor; DSM: Diagnostic and statistical manual of mental disorders; EMG: Electromyography; FDG: 18 F-Fluoro-deoxyglucose; GCP: Good clinical practice; GDNF: Glial cell-derived neurotrophic factor; GMP: Good manufacturing practice; IEC: Independent ethics committee; MDS: Movement disorder society; MMP: Matrix metalloproteinase; MMSE: Mini-mental state examination; MR: Magnetic resonance; MRI: Magnetic resonance imaging; MSC: Mesenchymal stem cells; NT-3: Neurotrophins-3; 6OHDA: 6-HydroxyDopamine; PD: Parkinson’s disease; PET: Positron emission tomography; PSP: Progressive supranuclear palsy; ROM: Range of motion; QC: Quality control; SD: Standard deviation; SEADL: Schwab and England activities of daily living; SPECT: Single photon emission computed tomography; UPDRS: Unified Parkinson’s disease rating scale.

## Competing interests

The authors declare that they have no competing interests.

## Authors’ contributions

RG contributed to study design and to write the clinical protocol; specifically she designed the pre-clinical study, contributed to set up the procedures for mesenchymal stem cell GMP validation, production and quality controls and wrote the protocols and performed the submission of the trial to the regulatory authorities. MC: contributed to study design and to write the clinical protocol; specifically he defined the procedure for patient selection, clinical evaluation and follow-up. MI contributed to study design and to write the clinical protocol; specifically he defined the procedure for cell administration. IUI contributed to study design and to write the clinical protocol; specifically he defined the procedure for the multifactorial movement analysis. TM contributed to design the pre-clinical study, set up the procedures for mesenchymal stem cell GMP validation and production. MV contributed to design the pre-clinical study, set up the procedures for mesenchymal stem cell GMP validation and quality control. EM contributed to set up the procedures for mesenchymal stem cell GMP validation and production. VB performed the ELISA assay and the real-time PCR studies. RB contributed to study design and to write the clinical protocol; specifically he defined the procedure for the PET analysis. AC took care of the bone marrow aspiration procedure and haematological patient assessment. NF took care of the bone marrow aspiration procedure and haematological patient assessment. LL contributed to study design and to the pre-clinical study. GP conceived the clinical trial, contributed to study design and to write the clinical protocol. All authors read and approved the final manuscript.
